# Development and Validation of a Nomogram for Predicting Blood Pressure Change Failure in Patients with Pheochromocytoma and Concomitant Hypertension after Adrenalectomy

**DOI:** 10.3390/jcm12030874

**Published:** 2023-01-22

**Authors:** Yuntian Ge, Yunhong Zhan, Chunyu Pan, Jia Li, Zhenhua Li, Song Bai, Lina Liu

**Affiliations:** 1 Department of Urology, Shengjing Hospital of China Medical University, Shenyang 110004, China; 2 Department of General Surgery, Shengjing Hospital of China Medical University, Shenyang 110004, China

**Keywords:** blood pressure change failure, nomogram, pheochromocytoma, adrenalectomy

## Abstract

(1) Background: Pheochromocytoma is a common cause of secondary hypertension, which is considered curable; nevertheless, some patients still suffer from hypertension after adrenalectomy. Therefore, we developed and validated a nomogram for predicting blood pressure change failure in patients with pheochromocytoma and concomitant hypertension after adrenalectomy. (2) Methods: The development cohort of this study consisted of 259 patients with pheochromocytoma who underwent adrenalectomy at our center between 1 January 2007 and 31 December 2018. Each patient’s clinicopathologic data were recorded. LASSO (the least absolute shrinkage and selection operator) regression was used to reduce and select the features of the data. Furthermore, we used multivariate logistic regression analysis to develop the prediction model. An independent cohort of 110 consecutive patients from 1 January 2019 to 31 December 2021 was used for validation. The performance of this nomogram was assessed with regard to discrimination, calibration, and clinical usefulness. (3) Results: 40.9% and 46.4% of patients experienced blood pressure change failure in the development and validation cohorts of this study, respectively. We found that older patients with a longer duration of hypertension and concomitant cardiovascular events were more likely to suffer from blood pressure change failure. In the validation cohort, the model manifested great discrimination with an AUROC (area under the receiver operating characteristic) of 0.996 (*p* < 0.001) and good calibration (unreliability test, *p* = 0.359). Decision curve analysis demonstrated that the model was clinically useful. (4) Conclusions: This study presented a reliable nomogram that facilitated individualized preoperative prediction of blood pressure change failure after adrenalectomy in patients with pheochromocytoma, which may help decision-making in perioperative treatment and follow-up strategies.

## 1. Introduction

Pheochromocytoma is a rare tumor that grows from chromaffin cells in the adrenal medulla. The incidence of pheochromocytoma is 0.6 cases per 100,000 person-years in the general population, and the prevalence is 0.1–0.6% in patients with hypertension [1,2,3,4]. It presents with extensive symptoms, such as headaches, sweating, palpitations, and hypertension due to hypersecretion of catecholamines. Hypertension is the most common clinical manifestation [5]. Sustained hypertension occurs in about 50–60% of patients, and 30% experience paroxysmal hypertension [6,7,8]. 

Adrenalectomy after preoperative medical preparation with α-blockade is the cornerstone of treatment, which has been shown to normalize blood pressure [9,10]. However, hypertension may persist after successful surgical intervention in half of the patients. Paolo et al. reported that only 46% of patients who were operated on for hormonally active adrenal tumors returned to the normal range of blood pressure after 77 months of follow-up, and age was the only significant predictor of long-term persistent hypertension in a retrospective study that involved 87 patients [11]. Pierre-François et al. also reported that up to half of the operated patients were still hypertensive after one year post operation in a single-center series retrospective analysis, which involved 70 pheochromocytoma patients with hypertension [12]. Previous studies addressing this issue are scarce, and the conclusions are controversial. Besides, most of these studies were published ten years ago with small sample sizes. 

A nomogram derived from a predictive model is accepted as a reliable tool for predicting risk by incorporating and illustrating important predictors of significant clinical outcomes [13]. To the best of our knowledge, there is no nomogram for predicting the risk of blood pressure change failure in patients with pheochromocytoma. Therefore, we developed and validated a model for preoperative prediction of blood pressure change failure in such patients after successful adrenalectomy in a large cohort.

## 2. Materials and Methods

### 2.1. Patients

**Figure 1:**
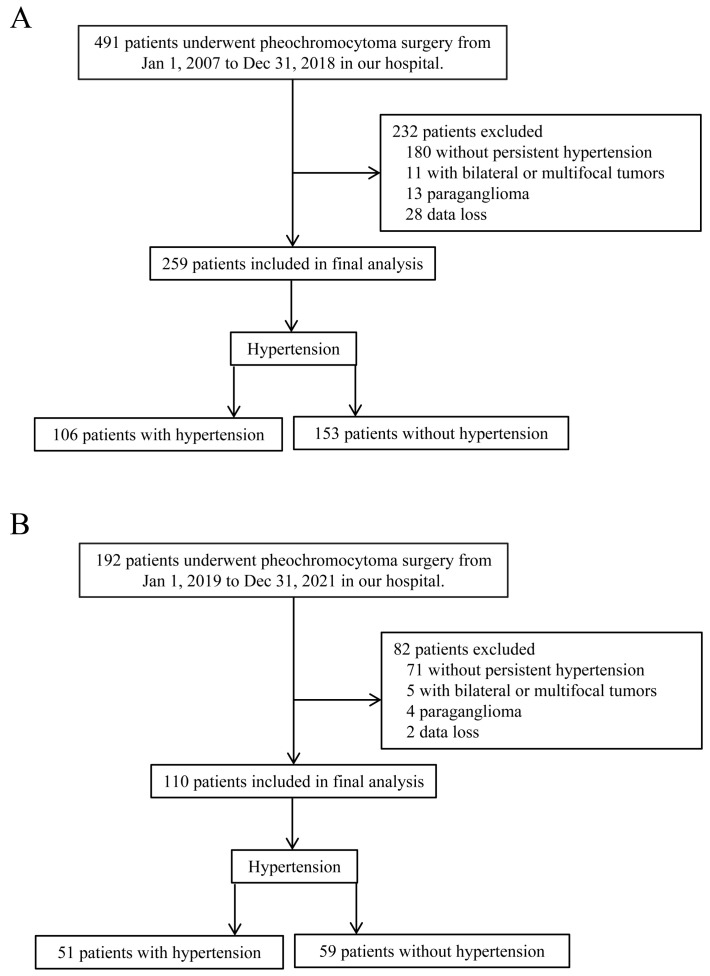
Flowchart of study. (**A**): Inclusion and exclusion flow chart of development cohort; (**B**): Inclusion and exclusion flow chart of validation cohort.

The development cohort of this study consisted of 259 patients who underwent pheochromocytoma adrenalectomy at our center between 1 January 2007 and 31 December 2018, while the validation cohort consisted of 110 consecutive patients from 1 January 2019 to 31 December 2021, who met the same inclusion and exclusion criteria. A flow chart illustrating the patient selection process is detailed in Figure 1.

Ethical approval (Ethical Committee No.2022PS724K) was offered by the Ethics Committee of Shengjing Hospital Affiliated to China Medical University in Shenyang, China. All the informed consent of these patients in this study has been obtained. 

### 2.2. Inclusion and Exclusion Criteria

Inclusion criteria: Those patients with persistent hypertension before surgery who underwent laparoscopic or open unilateral adrenalectomy (unilateral and solitary tumor), and whose pathologic results showed pheochromocytoma were finally included in this research. The clinical stage was localized (no adjacent organ invasion or metastasis). Exclusion criteria: Those with pheochromocytoma familial history, or malignancy were excluded from this study. 

### 2.3. Preoperative Preparation

The preoperative diagnoses were based on laboratory tests and diagnostic imaging. For patients who present typical biochemical manifestations of pheochromocytoma, α adrenoreceptor antagonists (doxazosin, terazosin, or prazosin) were conventionally used at least two weeks before the operation. Intravenous crystalloid or colloid fluid were admitted for volume expansion about three days before the operation. The criteria for preoperative medical preparation success included a blood pressure < 130/80 mmHg, a heart rate < 90/min, and a hematocrit < 0.45.

### 2.4. Characteristics and Outcome

Patient’s demographic characteristics (age, gender, and body mass index), comorbidities (duration of hypertension [from the onset of hypertension until the date of surgery], cardiovascular events, and diabetes mellitus), disease characteristics (tumor side, tumor size was measured by preoperative computed tomography, tumor enhanced CT (computed tomography) difference [between plain phase and arterial phase], types of α adrenoreceptor antagonists use [prazosin vs. others], use of crystalloid and colloid infusion, and 24-h urine vanillylmandelic acid/upper limits of normal value), intraoperative data (operation approach, intraoperative hemodynamic instability, and estimated blood loss and operation duration), and postoperative data (use of norepinephrine, postoperative transfusion) were recorded.

For each participant, preoperative blood pressure was measured three times at 2-min intervals after at least 5 min of rest in a seated position using a standardized automatic electronic sphygmomanometer (J30; Omron, Kyoto, Japan) before α-blockade treatment. Those with known hypertension and treated with anti-hypertension drugs were also recorded as having hypertension. Blood pressure was ascertained by self-report through telephone interview at three months postoperatively. The definition of hypertension was based on the 2003 World Health Organization International Society. Blood pressure change failure was defined as patients with a sustained BP of 140/90 mm Hg or more and those who still need to take antihypertensive drugs at three months postoperatively. Diabetes Mellitus was defined as a fasting blood glucose level greater than seven mmol/L or a non-fasting blood glucose level greater than 11 mmol/L. Cardiovascular events (CVE) were defined as proven myocardial infarction, angina pectoris, cerebral vascular accidents, and transient ischemic attacks [14]. The diagnosis of angina pectoris was based on the reporting of typical complaints by the patient with additional classical electrocardiogram changes indicating coronary insufficiency. Intraoperative hemodynamic instability (IHD) was defined as systolic pressure greater than 200 mmHg during operation, the mean arterial pressure less than 60 mmHg, or the use of norepinephrine to maintain the blood pressure to normal during surgery. The follow-up assessment was conducted using chart review and telephone calls. 

### 2.5. Statistical Analysis

IBM SPSS Statistics for Windows, version 22.0 (IBM Corporation, Armonk, NY, USA), STATA 15.0 (Stata Corporation, College Station, TX, USA), and R software (version 3.0.1; http://www.Rproject.org (accessed on 1 January 2022)) were used to analyze the data. In this investigation, the R packages “rms” and “glmnet” were utilized. The statistical significance levels presented were all two-sided, with statistical significance defined as a probability (*p*) value of less than 0.05. The median (interquartile range) is used to represent continuous variables. The independent samples Student’s *t*-test was used to compare the means of two continuous normally distributed variables. To compare two continuous non-normally distributed variables, the Mann–Whitney U-test was utilized. The number of categorical variables is provided (percentage). To compare categorical variables, the chi-squared and Fisher’s exact tests were used. 

The least absolute shrinkage and selection operator (LASSO) method was utilized to choose the most valuable predictive features from the primary data set in this study, which is suitable for decreasing high-dimensional data. Using the LASSO binary logistic regression model, all clinicopathologic variables were reduced to a small number of possible predictors based on 259 patients in the development cohort. There is no influence on the predicted regression parameters if the penalization coefficient lambda (λ) is big, but as it goes smaller, some coefficients may be decreased to near zero. We then used 10-fold cross-validation using minimum criteria and one standard error of the minimum criteria to find the best in the LASSO model (the 1-SE criterion). Finally, the model was re-fit using all of the non-zero coefficients selected by the Lasso method. 

The model’s performance was evaluated in an independent validation cohort. The validation cohort was given the logistic regression formula established in the development cohort, which was used to compute the likelihood for each patient. To quantify the model’s discrimination performance, the area under the receiver operating characteristic (AUROC) curve was measured. A 0.5 AUROC indicated no discrimination, whereas a 1.0 AUROC indicated perfect discrimination. The model’s calibration was evaluated using calibration plots, as well as the unreliability test and the Hosmer–Lemeshow (H-L) chi-square statistic (*p* > 0.05 indicates good calibration). Perfect calibration was indicated by a slope on the 45-degree line. By measuring the net benefits at different threshold probabilities in the validation cohort, decision curve analysis was used to determine the model’s clinical usefulness.

## 3. Results

After screening under the same inclusion and exclusion criteria, there were 259 patients enrolled in the development cohort and 110 patients in the validation cohort, respectively. The development cohort had a mean age of 56 years and a mean BMI of 23.5 kg/m^2^, and 55.38% were women, which was similar to the results in the validation cohort. There were 40.9% (100/259) of patients in the development cohort, while 46.4% (51/110) of patients in the validation cohort suffered from blood pressure change failure after successful surgery. (Table 1).

**Figure 2:**
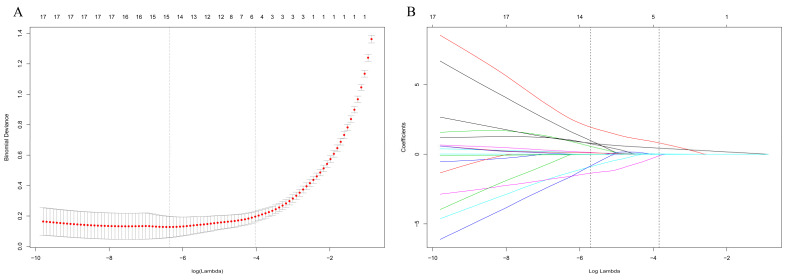
Texture feature selection using the least absolute shrinkage and selection operator (LASSO) binary logistic regression model. (**A**) The Tuning parameter (λ) for the LASSO model was selected using ten-fold cross validation via minimum criteria. Dotted vertical lines were drawn at the optimal values in reference to the minimum criteria and one s.e. of the minimum criteria. (**B**) A coefficient profile plot was produced against the log (λ) sequence. A vertical line was drawn at the value selected using ten-fold cross-validation.

**Figure 3:**
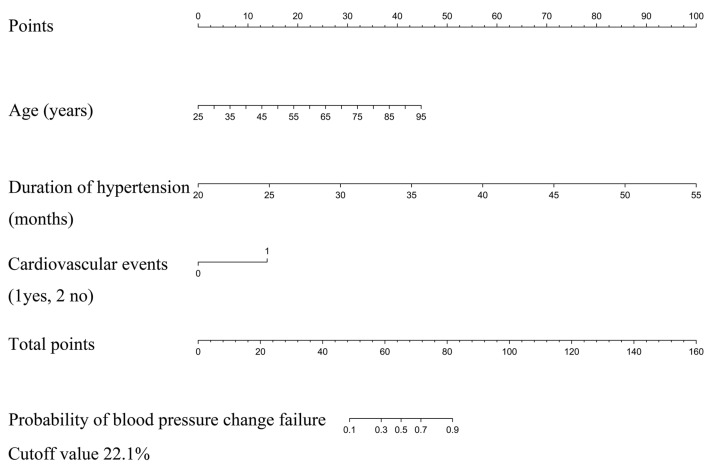
Nomogram to predict the probability of blood pressure change failure after pheochromocytoma surgery. Each clinicopathologic factor corresponds to a specific point by drawing a line straight upward to the point axis. After summing the points located on the total points axis, the sum represents the probability of blood pressure change failure in patients with pheochromocytoma after adrenalectomy.

In univariate analysis of the developed cohort, patients with older age, longer duration of hypertension, larger tumor size, higher VMA/upper limits of normal value, higher ratio of concomitant cardiovascular events and diabetes mellitus, and present in right side tumor were more likely to experience blood pressure change failure. Other candidate factors were not significantly associated with the outcome in this study (Table 2). Because the sample size in this study was insufficient to meet the recommended guidelines for each variable event, we used LASSO regression to filter variables for building the predicted model. Based on the results of LASSO regression, the potential predictors in the development cohort were reduced from 18 to three (Figure 2). The three variables with non-zero coefficients in the LASSO logistic regression model were presented in the final model (age, duration of hypertension, and concomitant cardiovascular events). Based on these results, we developed and validated a nomogram for predicting the probability of blood pressure change failure in patients with pheochromocytoma after adrenalectomy (Table 3 and Figure 3).

**Figure 4:**
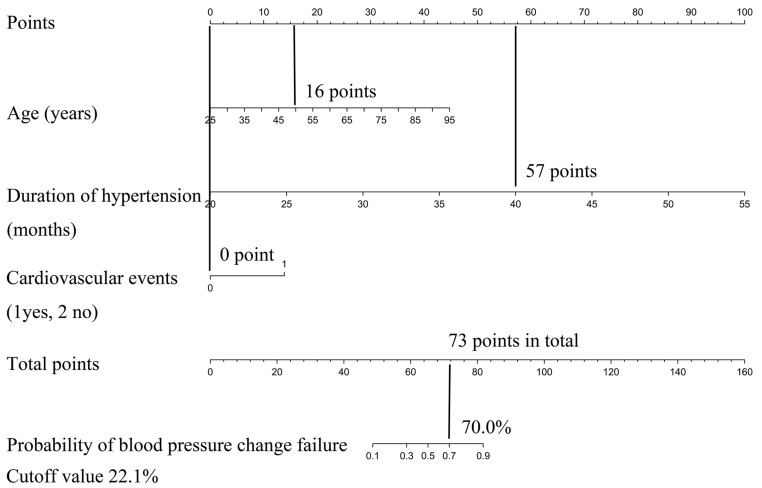
Nomogram to predict the probability of blood pressure change failure after pheochromocytoma surgery for example.

Each clinicopathologic feature corresponds to a specific point by drawing a straight line to intersect the point axis; after summing the points on the entire number line, the predicted probability of blood pressure change failure is obtained. For instance, a patient with the following features: 50 years old (16 points), 40 months of hypertension (57 points), and no concomitant cardiovascular events (0 points). The total points were 73, and the predicted probability of blood pressure change failure was approximately 70% (Figure 4). This calculated outcome could be used in decision-making for perioperative treatment and follow-up strategies.

**Figure 5:**
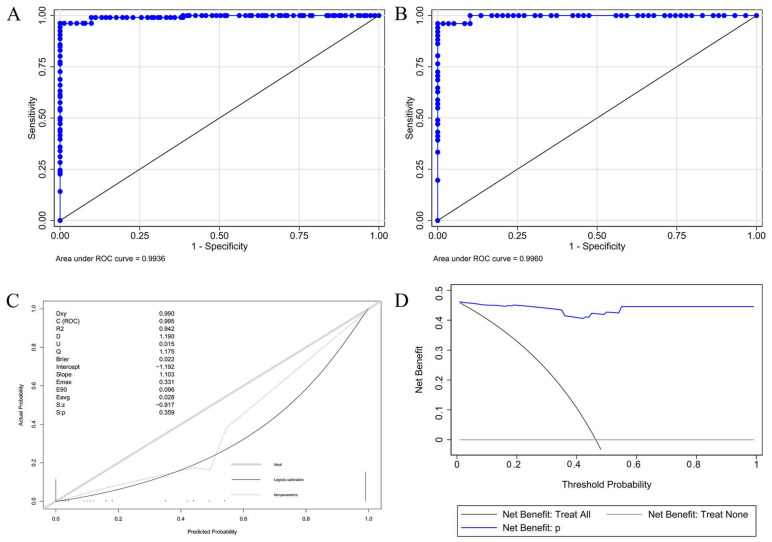
AUROC, calibration plot and decision curve analysis for the model. (**A**), AUROC in the development cohort; (**B**), AUROC in the validation cohort; (**C**), Calibration Plot shows the relationship between the predicted probabilities based on the nomogram and the actual values of the validation cohort. A plot along the 45-degree line would indicate a perfect calibration model in which the predicted probabilities are identical to the actual outcomes; and (**D**), Decision Curve Analysis: The *y*-axis measures the net benefit. The blue line represents the nomogram. The black line represents the assumption that all patients experience blood pressure change failure. The thin grey line represents the assumption that no patients experience blood pressure change failure. AUROC represents the discrimination ability of the model measured by the concordance index. Abbreviations: AUROC, area under the receiver operating characteristic curve.

The AUROC for the development cohorts and validation cohorts was 0.994 and 0.996. The cut-off value for the risk probability of this predicted model was 0.221, and the specificity and sensitivity were (1.000 and 0.962). The unreliability test statistic of calibration in this validation cohort was 0.015, the *p*-value was 0.359, Emax was 0.331, and Eavg was 0.028, indicating that the model was well-calibrated. This curve suggests that it would be more beneficial to use this nomogram to predict blood pressure change failure in patients after adrenalectomy. The net benefits are comparable across all ranges (Figure 5).

## 4. Discussion

Although pheochromocytoma has been considered a curable cause of secondary hypertension, half of the patients still suffer from continuous hypertension after successful adrenalectomy. Therefore, we developed and validated a nomogram for predicting the probability of blood pressure change failure in patients after adrenalectomy. In this study, 40.9% and 46.4% of patients experienced blood pressure change failure in the development and validation cohorts, respectively. Based on the results of LASSO regression, older patients with a longer duration of hypertension and concomitant cardiovascular events were more likely to suffer from blood pressure change failure. 

In previous studies, the prevalence of blood pressure change failure ranged from 30–70% after successful adrenalectomy [12,15,16]. In this study, 40.9% and 46.4% of patients experienced blood pressure change failure in the development and validation cohorts, respectively. The results are consistent with those of previous studies. 

This study revealed that older age at presentation was a significant predictor of blood pressure change failure in patients after adrenalectomy. In line with this, Paolo et al., in a retrospective study on 48 patients with benign adrenal tumors, found that 29% of patients still suffered from persistent hypertension that required antihypertensive drugs, and 25% of patients required less intensive hypertensive treatment than before adrenalectomy after 77 months of follow-up, and showed that aging was the only significant factor predictive of persistent hypertension [11]. In addition, Pradeep et al., in a prospective cohort study involving 26 patients with pheochromocytoma or paraganglioma, found that 33% of patients were still hypertensive and required antihypertensive drugs. This study also showed that older patients had a greater likelihood of persisting hypertension after successful adrenalectomy [15]. A possible reason could be that as patients get older, the ability to regulate inflammation becomes weaker, which leads to sustained tissue infiltration by leukocytes and the chronic release of proinflammatory cytokines and chemokines [17]. Subsequently, endothelial dysfunction due to these changes directly increases systemic vascular resistance, which leads to increased blood pressure. It is also the plausible etiology of essential hypertension [18].

Cardiovascular events are a common consequence of long-standing hypertension due to its role in the progression of arteriosclerosis [19,20]. In this study, patients with a longer duration of hypertension and concomitant cardiovascular events were more likely to suffer from blood pressure change failure after pheochromocytoma surgery. Paolo et al. also found that patients who had been hypertensive for five years or more had a higher probability of blood pressure change failure than those who had been hypertensive for less than five years, regardless of Conn’s and Cushing’s syndromes and pheochromocytoma [11]. The previous study also showed that with increasing duration of hypertension, individuals with primary aldosteronism lose the ability to reverse the structural vascular changes associated with secondary hypertension [21]. Arteriosclerosis induced by excess catecholamine and prolonged hypertension might explain this event, which is characterized by vascular remodeling and stiffening, subsequently causing persistent hypertension [22].

In this study, diabetes mellitus was not a predictor of blood pressure change failure in patients after pheochromocytoma surgery. However, Pradeep et al. found that a high ratio of patients concomitant with diabetes mellitus was observed in the blood change failure group (*p* = 0.04) [15]. This discrepancy may be due to the small sample size and heterogeneity of the criteria included in their study, in which patients were enrolled in both pheochromocytoma and paraganglion.

Pheochromocytoma is generally considered to be a curable cause of hypertension. However, in this study, blood pressure change failure was presented in nearly half of the patients with pheochromocytoma after successful adrenalectomy; thus, it should not be unreservedly considered as a surgically remediable cause of hypertension. The plausible explanation may be that patients are also likely to have non-catecholamine-induced hypertension, which may be essential or perhaps due to another underlying secondary cause. In addition, individuals with long-term hypertension lose the ability, with increasing age, to reverse the structural vascular changes associated with secondary hypertension [23]. Therefore, after successful adrenalectomy for pheochromocytoma, patients should be followed-up indefinitely, especially those with older age with a longer duration of hypertension and concomitant cardiovascular events. Based on this predictive model, during the perioperative period and postoperative follow-up duration, for these patients who would encounter blood pressure change failure following surgery, antihypertensive drugs should be given timely according to the level of postoperative blood pressure, rather than long-term supervision, to avoid hypertension-related complications. In addition, preoperative medical preparation strategies of α-blockade in combination with other antihypertensive medications may be more appropriate for patients who would experience blood pressure change failure following surgery.

This study has certain limitations. First, our study followed a retrospective design from a single center. Second, due to the long recruitment time, perioperative preparation strategies were diverse and non-standard, which might have influenced the outcome. Third, several potential factors were not included, such as genetic mutation data. Fourth, some factors related to blood pressure change after surgery were not considered, such as a family history of hypertension, which suggests the presence of a predisposition to essential hypertension [24]. Fifth, external validation in other cohorts is also needed to confirm the generality and clinical usefulness of the results. Sixth, due to the long recruitment time, urinary VMA excretion was selected as the diagnostic test in order to unify the criteria. Finally, the definition of hypertension duration may be misclassified since patients may not have detected hypertension for months or years. Nevertheless, this nomogram is the first model to predict the probability of blood pressure failure in patients with pheochromocytoma after surgery based on a large center and with an excellent temporal external validation. It may help in perioperative management and follow-up strategies, and may be of use to both patients and physicians to predict the risk efficiently.

## 5. Conclusions

Blood pressure change failure is presented in less than half of patients with pheochromocytoma after successful adrenalectomy; we identified three independent risk factors: older age with a longer duration of hypertension and concomitant cardiovascular events. This is a reliable nomogram that can facilitate the individualized preoperative prediction of blood pressure change failure after successful adrenalectomy in patients with pheochromocytoma, which may help in perioperative management and follow-up strategies. 

## 6. Patents

This section is not mandatory but may be added if there are patents resulting from the work reported in this manuscript.

## Figures and Tables

**Table 1:** Characteristics of patients in the development and validation cohort for this study.

Variables	Development Cohort	Validation Cohort
Number of patients (%)	259 (100)	110 (100)
**Demographic characteristics**		
Mean age (years)	56.0 (49.0, 66.0)	53.0 (49.0, 64.0)
Gender (male/female)	114 (44.0)/145 (56.0)	52 (47.3)/58 (52.7)
BMI (kg/m^2^)	23.5 (21.1, 25.4)	23.9 (21.1, 25.6)
**Comorbidities**		
Duration of hypertension (months)	23.0 (21.0, 33.0)	25.0 (20.0, 33.0)
Cardiovascular events (yes)	101 (39.0)	44 (28.8)
Diabetes Mellitus (yes)	107 (41.3)	45 (40.9)
**Preoperative data**		
Tumor side (left/right)	117 (45.2)/142 (54.8)	46 (41.8)/64 (58.2)
Tumor size (cm)	5.0 (3.9, 6.5)	5.0 (3.9, 6.0)
**Enhanced CT difference (Hu)**	41 (20, 65)	40.0 (21.0, 70.0)
Types of α adrenoreceptor antagonists (prazosin)(prazosinothers)	148 (57.1)	66 (60.0)
Use of crystalloid and colloid infusion (yes)	162 (62.54)	74 (67.3)
VMA/upper limits of normal value	1.9 (1.3, 2.8)	1.97 (1.38, 2.78)
**Intraoperative data**		
Operation approach (open/laparoscopy)	48 (18.5)/211 (81.5)	18 (16.4.)/92(83.6)
IHD	78 (30.1)	28 (25.5)
Estimated blood loss (ml)	200 (100, 500)	200 (100, 400)
Operation duration (minutes)	150 (95, 185)	141 (103, 184)
**Postoperative data**		
Use of NE	61 (23.6)	23 (20.9)
Postoperative transfusion	53 (20.5)	23 (20.9)

Continuous variables with normal distribution were reported as the mean ± standard deviation (SD); Non-normal continuous variables were expressed as median (interquartile range); Categorical variables were reported as number (percentage). Abbreviations: BMI, body mass index, CT, computed tomography; VMA, vanillylmandelic acid; IHD, intraoperative hemodynamic instability; and NE, norepinephrine.

**Table 2:** Univariate analysis of patients in the development and validation cohort for this study.

	Development Cohort 259 (100)			Validation Cohort 110 (100)		
Variables	Without Hypertension	Hypertension	*p*-Value	Without Hypertension	Hypertension	*p*-Value
Number of patients (%)	153 (59.1)	106 (40.9)		59 (53.6)	51 (46.4)	
**Demographic characteristics**						
Mean age (years)	52.0 (44.0, 61.0)	64.0 (52.0, 72.0)	<0.001	51.0 (44.0, 56.0)	64.0 (52.0, 71.0)	< 0.001
Gender (male/female)	68 (44.4)/85 (55.6)	46 (43.4)/60 (56.6)	0.867	27 (45.8)/32 (52.4)	25 (49.0)/26 (51.0)	0.733
BMI (kg/m^2^)	23.4 (21.3, 25.3)	24.2 (20.8, 25.6)	0.827	24.2 (21.5, 25.4)	23.4 (20.8, 25.8)	0.442
**Comorbidities**						
Duration of hypertension (months)	21.0 (19.0, 23.0)	36.0 (32.0, 41.0)	<0.001	21.0 (17.0, 23.0)	34.0 (31.0, 40.0)	0.001
Cardiovascular events (yes)	44 (28.8)	57 (53.8)	<0.001	20 (33.9)	29 (56.9)	0.017
Diabetes Mellitus (yes)	47 (30.7)	60 (56.6)	<0.001	18 (30.5)	27 (52.9)	0.018
**Preoperative data**						
Tumor side (left/right)	80 (52.3)/73 (47.7)	37 (34.9)/69 (65.1)	0.006	31 (52.5)/28 (47.5)	15 (29.4)/36 (70.4)	0.015
Tumor size (cm)	4.7 (35, 6.2)	5.2 (4.3, 7.0)	<0.001	4.3 (3.1, 5.9)	5.2 (4.5, 6.5)	0.004
**Enhanced CT difference (Hu)**	40.0 (21.0, 65.0)	45.0 (20.0, 79.0)	0.079	40.0 (21.0, 65.0)	45.0 (20.0, 79.0)	0.285
Types of α adrenoreceptor antagonists (prazosin vs. others)	89 (58.2)	59 (55.7)	0.548	40 (67.8)	26 (51.0)	0.074
Use of crystalloid and colloid infusion	98 (64.1)	64 (60.4)	0.368	40 (67.8)	34 (66.7)	0.900
VMA/upper limits of normal value	1.57 (1.05, 2.38)	2.29 (1.87,3.71)	<0.001	1.59 (1.22, 2.32)	2.24 (1.70,3.72)	0.008
**Intraoperative data**						
Operation approach (open/laparoscopy)	32 (20.9)/121(79.1)	16 (15.1)/90 (84.9)	0.238	10 (16.9)/49 (83.1)	8 (15.7)/43 (84.3)	0.858
IHD (yes)	41 (26.8)	37 (34.9)	0.163	10 (16.9)	18 (35.3)	0.030
Estimated blood loss (ml)	200 (100, 500)	200 (100, 600)	0.683	200 (100, 300)	300 (100, 600)	0.041
Operation duration (minutes)	150 (100, 195)	143 (92, 175)	0.586	140 (100, 184)	160 (103, 185)	0.243
**Postoperative data**						
Use of NE	39 (25.5)	20 (20.8)	0.378	14 (23.7)	9 (17.6)	0.435
Postoperative transfusion	32 (20.9)	21 (19.8)	0.829	11 (18.6)	12 (23.5)	0.531

Continuous variables with normal distribution were reported as the mean ± standard deviation (SD); Non-normal continuous variables were expressed as median (interquartile range); Categorical variables were reported as number (percentage). The student’s *t*-test was used to compare means of two continuous normally distributed variables and the Mann–Whitney U-test was used to determine means of two continuous non-normally distributed variables. The chi-squared test or Fisher’s exact test were used for categorical variables. Abbreviations: BMI, body mass index, CT, computed tomography; VMA, vanillylmandelic acid; IHD, intraoperative hemodynamic instability; and NE, norepinephrine.

**Table 3:** Multivariate binary logistic regression of patients in development cohort.

Variables	β (95% CI)	OR (95% CI)	*p*
Constant	−33.068 (−49.128, −17.009)	4.35 × 10^−15^ (4.61 × 10^−22^, 4.10 × 10^−8^)	
Mean age (years)	0.145 (0.008, 0.282)	1.156 (1.009, 1.324)	0.037
Duration of hypertension (months)	1.132 (0.493, 1.771)	3.103 (1.637, 5.883)	0.001
Cardiovascular events (yes versus no)	2.843 (0.011, 5.675)	17.168 (1.011, 291.414)	0.049
**Area under ROC curve**	**AUROC 95% CI**		
Development cohort	0.994 (0.986, 1.000)		<0.001
Validation cohort	0.996 (0.990, 1.000)		<0.001

The β coefficient, odds ratio, and 95% confidence interval were analyzed by multivariate binary logistic regression. OR, odds ratio; CI, confidence interval; ROC, receiver operating characteristic curve; AUC area, area under ROC.

## Data Availability

The data are not available and has been used is confidential.
